# Effect of Antioxidants on the Fibroblast Replicative Lifespan *In Vitro*

**DOI:** 10.1155/2020/6423783

**Published:** 2020-09-23

**Authors:** Izabela Sadowska-Bartosz, Grzegorz Bartosz

**Affiliations:** ^1^Department of Analytical Biochemistry, Institute of Food Technology and Nutrition, College of Natural Sciences, Rzeszow University, Zelwerowicza Street 4, 35-601 Rzeszow, Poland; ^2^Department of Bioenergetics, Food Analysis and Microbiology, Institute of Food Technology and Nutrition, College of Natural Sciences, Rzeszow University, Zelwerowicza Street 4, 35-601 Rzeszow, Poland

## Abstract

Replicative senescence is an unalterable growth arrest of primary cells in the culture system. It has been reported that aging *in vivo* is related to the limited replicative capacity that normal somatic cells show *in vitro*. If oxidative damage contributes to the lifespan limitation, antioxidants are expected to extend the replicative lifespan of fibroblasts. This article critically reviews the results of experiments devoted to this problem performed within the last decades under conditions of *in vitro* culture. The results of studied are heterogeneous, some papers showing no effects of antioxidants; most finding limited enhancement of reproductive capacity of fibroblasts, some reporting a significant extension of replicative lifespan (RLS). Both natural and synthetic antioxidants were found to extend the RLS of fibroblasts, either by a direct antioxidant effect or, indirectly, by activation of signaling pathways and activation of proteasomes or hormetic effects. Most significant prolongation of RLS was reported so far for nicotinamide, *N*-hydroxylamines, carnosine and Methylene Blue. These results may be of importance for the design of skin-protecting cosmetics.

## 1. Introduction

The skin is the largest and the most visible organ of the human body. Aged skin is biologically characterized by the flattening of the dermal-epidermal junction and a general atrophy of the extracellular matrix (ECM) with disorganized and reduced collagen and elastin. Fibroblasts are one the most important cellular components of the skin derma. In the skin, fibroblasts are responsible for the whole range of various functions. They include both homeostasis of the dermal ECM and maintenance of the physiological condition of other skin layers. The former is carried out by remodeling and renewing the ECM through degradation of used up dermal components and synthesis of new ones, predominantly collagen and elastin. During aging, skin fibroblasts undergo substantial changes in their morphology, functional activity, and proliferative potential. The number of dermal fibroblasts decreases with aging; this phenomenon, along with impairment of functional capacities of the remaining cells results in decreased ability to synthesize active soluble factors and to maintain proteostasis of components of the ECM. The skin thinning, the loss of skin flexibility and elasticity, and wrinkle formation, hallmarks of skin aging, are natural consequences of such a decline [1].

Fibroblasts have been the first and most broad cells cultured *in vitro*. Initially, it was believed that fibroblasts are immortal in *in vitro* culture [2], but later on, Hayflick demonstrated that their rRLS is limited. Hayflick and Moorhead found that human fibroblasts grown *in vitro* eventually reach a senescent condition (Phase III) after a long period of normal growth (Phase II) from the establishment of the primary culture (Phase I). At stage III, cells are arrested at the G1 phase of the cell cycle and express a specific morphological and biochemical phenotype [3, 4]. Telomere shortening is believed to be an intrinsic mechanism that limits the cellular doubling capacity, at least in human cells [5], although telomerase-independent mechanisms can contribute [6–8]. In human somatic cells, telomeres are eroded by almost 30–200 bp by each DNA replication [9]. When foreskin fibroblasts were transfected with vectors encoding the human telomerase catalytic subunit, in contrast to control clones, which showed telomere shortening and senescence, the telomerase-expressing clones had elongated telomeres, divided vigorously, and showed reduced staining for acidic *β*-galactosidase. The telomerase-expressing clones had a normal karyotype and exceeded their normal lifespan by at least 20 doublings [10].

Telomere shortening and/or telomere attrition is probably recognized as DNA damage, inducing a DNA damage checkpoint response [11]. Without being repaired, shortened telomeres cause continuous activation of the p53-growth inhibitory pathway and therefore drive cells into a state of irreversible growth arrest. Increased histone acetylation evoked by histone deacetylase (HDAC) inhibitors has been reported to induce senescence-related phenotypes in cultured human cells [8]. An increase in histone acetylation may be due to decreased nicotinamide adenine dinucleotide- NAD^+^-dependent HDAC (N-HDAC) activity in aging fibroblasts [12]. Interestingly, the protein Sir2, a gene silencer and positive longevity regulator in yeast, has been found to be N-HDAC [13].

A fundamental question is if all fibroblasts age replicatively at the same rate or if there is an inherent asymmetry in the replicative potential of dividing cells. While a tacit assumption of most of the studies is that the replicative potential is evenly distributed between daughter cells, there are some hints that it may not be the case. The variability of the results of *in vitro* longevity studies can be explained on the basis of the commitment theory of cellular aging. This theory implies that stem-like cells that are not committed to senescence are present in early passage fibroblast populations, but they give rise to committed cells with finite lifespan. During subsequent subcultivations, the uncommitted cells are diluted stochastically and populations containing less or no uncommitted cells have shorter RLS. According to this theory, final cell populations may be single cell clones [14–16].

To our knowledge, no published review has assembled research devoted to effect of antioxidants on the fibroblast RLS *in vitro*. This study is aimed at summarizing the available data on this subject.

## 2. Senescence-Related Changes in Fibroblasts

Morphological changes accompanying replicative aging of fibroblasts concern a failure to line up and appearance of irregularities of size and shape including increase in cell size, enlargement and flattening of the cell soma, increase of the nuclear volume [17], accumulation of granular material in the cytoplasm and cell debris in the medium [3, 4], chromatin decondensation [18], development of aneuploidy [4] and polyploidy [19], loss of response to growth factors, decreased activity of cell-cycle-dependent enzymes [20], reduced saturation density, and increased level of DNA damage, including oxidative damage and strand breaks [21, 22]. Acidic (senescence-associated) *β*-galactosidase (staining for activity at pH 6.0; SA-*β*-gal) is increased [23] and is commonly used as a marker of fibroblast senescence.

Human fibroblasts arrest in the G1 phase of the cell cycle upon entering the senescent state [24]. G1 arrest of senescent fibroblasts is irreversible, and the cells do not respond to mitogenic signaling anymore. The level of reactive oxygen species (ROS) was reported to increase significantly in senescent (55 population doubling; PD) vs. young (18 PD) fibroblasts as estimated with 2′,7′-dichlorofluorescin diacetate (DCFDA) [25]. Using the same fluorogenic probe, Kang et al. found an approximately eightfold increase in the ROS level between cells at PD38 and PD83 [26]. This increase in the ROS level was not accompanied by any major changes in the Raf/MEK/ERK pathway with aging but was attributed to a constitutive activation of Jun kinase in senescent fibroblasts [25].

The expression of a number of genes is altered during fibroblast aging *in vitro*. Many of them, including *p16*, *p21*, *EF1a*, procollagen a1 (III), and fibronectin, exhibit a dramatic change in expression at the terminal phase of cell aging [27–29]. The gene for PI3K IIb (a subtype of the PI3K family lipid/protein kinases) is upregulated by aging and downregulated by growth stimulation [12]. The accumulation of rhodamine 123 increases with increasing population PD, especially close to the end of RLS. The activity of aconitase, a FeS enzyme sensitive to oxidative stress, decreases [30]. Protein carbonyl content increases in senescent fibroblasts [31], although one study reported a decrease in this content [22].

## 3. The Effect of Antioxidants on the Replicative Lifespan of Fibroblasts

A hypoxic atmosphere was found to increase the RLS of fibroblasts *in vitro*. The replicative lifespan of IMR-90 cells cultured under 3% O_2_ was 142% and 157% (in two independent experiments) of that under 20% [32]. On this basis, it was expected that antioxidants would exert a similar effect.

One of the first papers concerning the effect of antioxidants on the fibroblast RLS reported a considerable prolongation of RLS by *vitamin E* (DL-*α*-tocopherol): 10 *μ*g/ml and 100 *μ*g/ml vitamin E increased RLS of WI-38 fibroblasts from 65 to 109 and 115 PD, respectively. The authors ascribed this effect to the restoration of vitamin E level typical of blood plasma (cell culture medium containing 10% fetal calf serum has about 10% of normal vitamin E level of blood plasma/serum). Removal of vitamin E after 73 PD did not attenuate the RLS prolongation [33]. However, the same authors were not able to reproduce this effect in subsequent experiments involving 19 subcultivations. In one set of experiments, control cells attained 57.7 ± 4.0 PD, while cells treated with 10 *μ*g/ml vitamin E reached 51.0 ± 6.0 PD. In another set, the number of PD was 51.0 ± 5.8 and 50.1 ± 2.7 for control cells and cells supplemented with 50 *μ*g/ml vitamin E, respectively. In experiments involving addition of 100 *μ*g/ml vitamin E, the PD values were 65.7 ± 13.2 for control cells and 67.8 ± 11.7 for vitamin E-treated cells. The authors link this difference to the use of different batches of serum [34].

Another main physiological antioxidant vitamin, *vitamin C* (ascorbic acid), was found to dose-dependently extend RLS of human embryonic fibroblasts, by 2, 6, and 11 PD at 2, 20, and 200 *μ*M ascorbic acid, respectively, when present from 40^th^ PD. Ascorbic acid decreased also SA-*β*-gal staining and ROS level estimated with DCFDA and dihydrorhodamine 123, increased the activity of aconitase, and decreased the levels of p53, phospho-p53 (phosphorylated at Ser15), and p21 proteins and formation of apurinic/apyrimidinic sites and relieved senescence-related G1 arrest [35].

Ascorbic acid phosphoric ester, phosphorylated at the 2,3-enediol moiety of ascorbic acid, is a stable form of ascorbic acid providing a continuous effect with low cytotoxicity compared with ascorbic acid [36]. Ascorbic acid phosphate (200 *μ*M) increased RLS of two lines of normal fibroblasts and a fibroblast line derived from a Werner syndrome patient by 3.5-8.5 PD and decreased the rate of telomere shortening. Treatment with ascorbic acid phosphate reduced the level of ROS detected with DCFDA in cells grown under 20% oxygen although it did not affect the ROS level in cells grown under 2% oxygen [37].

*Resveratrol* (3,5,4′-trihydroxystilbene), a polyphenol found in grape skin and thus in wine, preventing age-related diseases in rodents and extending lifespan in several species, has been suggested to be a calorie restriction mimic although this view has been questioned [38]. This compound has been reported to activate sirtuins (e.g., human SIRT1) via an indirect mechanism [39]. Nevertheless, resveratrol was found to cause cell cycle arrest and even apoptosis at higher doses. For example, it was shown that 6.25–12.5 *μ*M resveratrol blocked cell cycle, while 25 *μ*M resveratrol caused apoptosis in vascular smooth muscle cells [40]. In cancer cells, resveratrol caused irreversible cell cycle arrest and appearance of senescent morphology [41]. The physiological level of resveratrol in human blood can reach a concentration of approximately 2 *μ*M [42, 43]. Resveratrol concentrations higher than 10 *μ*M have a cytostatic effect [44]. Resveratrol was reported to be unstable in cell culture medium. In Basal Modified Eagle's Medium (BMEM), 96% of resveratrol was degraded and approximately 90 *μ*M H_2_O_2_ was generated, when 200 *μ*M resveratrol was incubated in BMEM at 37°C for 24 h. It was noticed that the degradation of resveratrol can be avoided by withdrawing sodium bicarbonate from the medium [45]. In another study, however, resveratrol was found to be quite stable in a cell culture medium, ca 75% of the initial concentration remaining after 48 h. In that study, 5 *μ*M resveratrol was found to slightly prolong RLS of fibroblasts (by 2 population doublings) [44]. In a different study, 65% of resveratrol was degraded in DMEM medium after 7 days; this degradation could be prevented by the addition of superoxide dismutase and pyruvate to the culture medium. In that study, resveratrol, as well as oxyresveratrol (a naturally occurring resveratrol analog present in mulberry *Morus alba* L. and acetyl resveratrol (another naturally occurring resveratrol analog found in marine invertebrates), decreased RLS of human Hs68 fibroblasts at concentrations of 1-20 *μ*M, even when these antioxidants were not degraded [46]. Other authors found that resveratrol (0.2 and 1 *μ*M) changed the expression of 47 genes; the changes included a decrease in the level of *INK4a*, cyclin-dependent kinase (*CDK*) and *α*2-macroglobulin mRNA. However, the compound did not affect RLS [47]. In our experiments, resveratrol as well as curcumin (1 *μ*M) decreased the RLS of H8F2p 25LM fibroblasts (unpublished).

Flavonoids are a large group of natural antioxidants, ubiquitously present in plants. They are present in fruits, vegetables, nuts, and plant-derived beverages such as tea and wine. *Quercetin* is the main flavonol present in our diet. It is the aglycon form of a number of other flavonol glycosides such as rutin (quercetin-3-*O*-rutinoside) and quercitrin (quercetin 3-*O*-a-L-rhamnoside). It is the most active flavonoid with the highest reported antioxidant activity and multiple mechanisms of antioxidant action. *Quercetin caprylate* (concentration not specified but ≤5 *μ*g/ml) was found to increase the RLS of human foreskin fibroblasts (HFF1); this increase was less than 5%, but statistically significant. Quercetin and quercetin caprylate maintained young morphology of fibroblasts, delayed the appearance of senescent phenotype, increased their growth rate, and had a morphologically rejuvenating effect on terminally senescent fibroblasts. They also activated proteasomes, and this effect was suggested to be the main one responsible for the antiaging effect of these compounds on the fibroblasts [48]. Recent studies evidenced that quercetin and caffeic acid in old fibroblasts induced higher expression of aging counteracting genes compared to resveratrol; caffeic acid increased sirtuin 6 (*SIRT6*) gene expression, and quercetin increased sirtuin 1 (*SIRT1*) gene expression, thus inducing an antiaging effect. Moreover, quercetin as well as caffeic acid induced higher expression of *SIRT1* and Nuclear Receptor Subfamily 1, Group D, Member 1 (*NR1D1*) genes than resveratrol in fibroblasts [49].

*Epigallocatechin gallate* (EGCG) is the most active and major polyphenol in green tea and is well known to be a primary contributor to the potential benefits of green and black tea to human health [50, 51]. EGCG has a very similar structure to that of resveratrol, including hydroxyls in the two metapositions of the A ring, *trans* to the B-ring with a 4′ or 3′,4′-hydroxyl pattern [52]. EGCG was reported to increase the activity of antioxidant enzymes, including superoxide dismutases and catalase, and decreased the ROS level in replicatively advanced fibroblasts although its effect on their RLS was not reported [53].

Other flavonoids, anthocyanidins, were demonstrated to be effective in prolonging the RLS of fibroblasts *in vitro*. *Cyanidin* (concentration not specified) prolonged the RLS of normal human diploid fibroblasts (WI-38) by 4 PD (from 64 to 68) when added at passage 26, by 3 PD when added at passage 42, and by 1 PD when added at passage 58 [54]. *Malvidin*, one of the most abundant polyphenol components of black rice and red wines, had a similar effect. When added at passage 26, malvidin prolonged RLS from 64 to 67 PD; when added at passage 42, it prolonged RLS from 42 to 46 PD; and when added at 58 PD, it prolonged RLS of WI-38 fibroblasts from 62 to 63 PD. Unfortunately, malvidin concentration was also not specified [55].

Liu et al. indicated that *urolithin A*, a product of ellagitannin degradation by gut bacteria, significantly increased the expression of type I collagen and reduced the expression of matrix metalloproteinase 1 (MMP-1) in senescent human dermal fibroblasts. What is more, urolithin A decreased intracellular ROS, which can be partially due to the activation of the Nrf2-mediated antioxidant response. Urolithin A (50 *μ*M) caused changes in cell morphology and inhibited cell proliferation, due to cell cycle arrest in the G2/M phase. Nevertheless, SA-*β*-gal staining and *γ*H2AX immunofluorescence staining showed that cellular senescence status of the fibroblasts did not change [56].

*18α-Glycyrrhetinic acid* (enoxolone), a pentacyclic triterpenoid derived from the hydrolysis of glycyrrhizic acid, was obtained from the herb liquorice *Glycyrrhiza glabra*. It is used in flavoring, and it masks the bitter taste of drugs like aloe and quinine. It is effective in the treatment of peptic ulcers and has antitussive and possibly antiviral, antifungal, antiprotozoal, and antibacterial activities. This compound, present in the culture of lung fibroblasts, increased their RLS from 58.5 to 64.3 PD. It decreased ROS production, but its main effect is supposed to be stimulation of the synthesis of proteasome subunits mediated by nuclear erythroid factor 2 (Nrf2). Knockdown of *Nrf2* by siRNA decreased the RLS of fibroblasts (from 44.7 down to 42.5 PD) [57].

*Berberine*, an isoquinoline alkaloid compound, which is extracted from numerous herbs across the globe, has a long history of medicinal use in China as well as in traditional Ayurvedic medicine. Berberine improved the morphology and induced rejuvenation of replicatively advanced human lung fibroblasts, as judged from senescence markers including SA-*β*-gal. Berberine promoted the entry of cell cycles from the G0 or G1 phase to S/G2-M phase thus improving cell growth and proliferation. Enhanced proliferation of 2BS (PD45) and WI38 (PD45) cells was observed during seven days of incubation. Only 3.5% SA-*β*-gal-positive 2BS cells were seen in the treated PD30 cells, while 60% were observed in late PDL cells. In berberine-treated 2BS cells (PD45), the SA-*β*-gal-positive cell rate reverted to the level of young cells (16%), as compared to the control group (56.5%). The level of p16 decreased and that of cyclin protein and cyclin-dependent kinases, such as cyclin D1 and CDK4, increased. The level of phosphorylated retinoblastoma protein (pRB) was upregulated [58].

*Betel vine* (*Piper betle* Linn. (PB)) extract, a traditional medicine that is widely used to treat different diseases in Asia, modulated the expression of senescence-associated genes of senescent human fibroblasts. *Piper betle* extracts at 0.4 mg/ml could improve cell proliferation of young, presenescent, and senescent fibroblasts by 143%, 127%, and 157%, respectively. Increased expressions of *PRDX6*, *TP53*, *CDKN2A*, *PAK2*, and mitogen-activated protein kinase 14 (*MAPK14*) were observed in senescent fibroblasts compared to young and presenescent cells. Treatment with the extracts modulated the transcriptional profile changes in senescent HDFs. Expression of Cu,Zn-superoxide dismutase (*SOD1*) gene increased, whereas those of *GPX1*, *PRDX6*, *TP53*, *CDKN2A*, *PAK2*, and *MAPK14* decreased in senescent treated with the extract compared to untreated senescent fibroblasts [59].

*Ginsenoside Rg3*, enriched from steamed or heated ginseng roots, exerts multiple pharmacological effects showing anticancer, antiangiogenic, and antidiabetic activities [60]. It was found that Rg3(*S*) reverses the replicative senescence of fibroblasts by modulating Akt-mTOR-sirtuin signaling to promote the biogenesis of mitochondria. Ginsenoside stereoisomer Rg3(*S*) (10 and 30 *μ*M) decreased SA-*β*-gal staining and restored the decreased ATP level, the decreased NAD^+^/NADH ratio, and the increased ROS level in senescent fibroblasts. Rg3(*S*) downregulated phosphatidylinositol 3-kinase/Akt through the inhibition of mTOR by cell cycle regulators like p53/p21 in senescent HDFs, whereas the stereoisomer Rg3(*R*) did not alter the corresponding signaling pathways. Rg3(*S*) stimulated mitochondrial biogenesis via activation of sirtuin 3/PGC1*α* [61].

Expression of senescence-associated genes related to antioxidants and DNA damage-associated signaling, insulin/insulin-like growth factor-1 signaling, cell differentiation, and cell proliferation pathways was modulated by *Chlorella vulgaris extract* in the course of replicative senescence of human fibroblasts. Treatment of young HDFs with *Chlorella vulgaris* extract decreased the expression of *SOD1*, catalase (*CAT*), and copper chaperone for superoxide dismutase (*CCS*) genes and increased the expression of the *SOD2* gene. Treatment of senescent fibroblasts with *C. vulgaris* extract downregulated also the expression of genes for the p53 protein and cyclin-dependent kinase inhibitor 2A and upregulated the expression of *MAPK14* gene in presenescent and senescent fibroblasts [62].

*Kinetin*, a synthetic cytokinin plant hormone, which has some senescence-retarding effects in plants and acts as an indirect antioxidant, stimulated antioxidant defense not only in plants [63] but also in animal cells, including fibroblasts [64]. Kinetin (40-200 *μ*M) treatment of cultured fibroblasts did not have any significant effect on RLS, although the cells retained some characteristic features of young cells like a lower level of fluorescence due to accumulation of lipofuscin, lack of highly polymerized and rod-like actin filaments, and lack of multiple and disorganized microtubular network [65].

The dipeptide *carnosine* (*β*-alanyl-L-histidine) is an antioxidant and compound preventing nonenzymatic protein glycosylation (glycation), present in muscle cells at a concentration of ca 20 mM. Its effect on fibroblast aging and RLS was thus tested at high concentrations (20-50 mM). 20 mM carnosine added starting from passage 9 extended the RLS of human foreskin HFF1 fibroblasts by 7.4 and 9.4 PD, while 30 mM carnosine present from passage 13 extended it by 3.0 and 6.3 PD; 50 mM carnosine added at passage 10 did not prolong RLS although it extended chronologic lifespan (from 432 to 716 days). In human fetal lung fibroblast cells (MRC-5), 20 mM carnosine present from passage 14 extended RLS by 10 and 14 PD in independent experiments, while 30 mM carnosine present from passage 14 extended RLS by 4 to 8 PD. Interestingly, the transfer of MRC-5 fibroblasts at 55 PD, which already showed characteristic signs of senescence, to a medium containing 20 mM or 30 mM carnosine, induced a remarkable morphological rejuvenation and RLS prolongation, by 13.0 and 12.1 PD, respectively. The effect of L-carnosine on cell morphology is much stronger in DMEM medium than in MEM medium. D-Carnosine (20 mM) did not increase the RLS of HFF1 fibroblasts, in contrast to L-carnosine, although the antioxidant properties of both forms of carnosine did not differ. This suggests that RLS prolongation by L-carnosine is due to some features of this compound other than its antioxidant action [66]. Hipkiss and Brownson proposed that carnosine may react with protein carbonyl groups to produce protein-carbonyl-carnosine adducts (“carnosinylated” proteins). The authors suggested that these proposals can help explain the antiaging actions of carnosine and its presence in nonmitotic cells of long-lived mammals [67]. Furthermore, Shao et al. showed that human diploid fetal lung fibroblasts grown in 20 mM carnosine exhibited a slower telomere shortening rate and extended lifespan measured in population doublings. It seems that the reduction in telomere shortening rate and damages in telomeric DNA made a significant contribution to the antiaging effect of carnosine [68].

*Nicotinamide*, a precursor of the coenzyme NAD^+^, serves to rapidly synthesize NAD^+^ through the salvage pathway once taken up by cells. It inhibits sirtuin activity. It has been shown to positively affect cell survival in a variety of cell types. It also enhances an adaptive response to physical and chemical damage, protects brain cells from oxidative damage caused by reperfusion after ischemic infarction, and prevents injury of pancreatic islet cells during free radical exposure. Moreover, it was reported to protect against mitochondrial damage [26]. Nicotinamide (0.5 and 5 mM) slightly increased RLS of human diploid foreskin fibroblasts (FB0603) (from 23 to 25 PD) [47]. In another study, 3 mM nicotinamide was found to induce transient and reversible morphological rejuvenation of aged IMR-90 fibroblasts. Nicotinamide increased the activity of histone acetyltransferase and attenuated the fibroblast-aging related changes in the expression of the gene coding for PI3K IIb (a subtype of the PI3K family lipid/protein kinases) [12]. Kang et al. found a significant effect of 5 mM nicotinamide, dependent on the duration of treatment. Nicotinamide was introduced at PD 30. When the compound was withdrawn at PD 90 from cells grown in high glucose (25 mM), the cells proliferated for 21-more doublings and senesced at PD 111, while when nicotinamide was removed at PD 74, they proliferated until PD 93. The cells cultured in the normal glucose medium (5.5 mM glucose) proliferated significantly further after the removal of nicotinamide (cells senesced at PD 121, when removed at PD 100, and at PD 110 when removed at PD 86). Apparently, cell proliferation stopped at 20 or so PDs after the removal of nicotinamide. The cells that were cultured in the high-glucose medium proliferated until PD 109 when fed nicotinamide starting from PD 52, while those fed starting from PD 70 proliferated only to PD 79. Therefore, the earlier the cells were treated with nicotinamide, the more the lifespan was extended. For the cells cultured in normal level glucose, longer-term nicotinamide treatment was necessary to be effective as delayed treatment showed no effect. The authors argued that these results indicate that lifespan extension was induced not by nicotinamide itself but by certain changes caused by nicotinamide and the effect of this change lasted for a substantial number of population doublings [26]. Kwak et al. found that 5 mM nicotinamide prolonged the RLS of human foreskin fibroblasts, decreased appearance of SA-*β*-gal activity, lipofuscin accumulation, and mitochondrial superoxide level [69].

A procedure simulating calorie restriction, viz. culturing fibroblasts in a medium of lowered glucose concentration, was found to increase RLS of human fibroblasts. This effect has been ascribed to an increase in the activity of nicotinamide phosphoribosyltransferase, elevation in the intracellular NAD^+^ level, and activation of sirtuin1 activity [70].

*α-Phenyl-t-butyl nitrone* (PBN), which can act as an antioxidant, increased dose dependently the RLS of the human diploid fibroblast strain IMR-90, in the concentration range of 100-800 *μ*M. *α*-Phenyl-t-butyl nitrone was most effective, when added to cells just before they reduced their growth rate. The treatment of replicatively young cells with PBN caused a small inhibition of cell growth. An optimal effect (increase in RLS by 7.1 PD) was observed when PBN was added at 42.2 PD; addition at 21.1 PD increased RLS only by 0.9 PD [22]. *N-t-butyl hydroxylamine*, a product of PBN decomposition, prolonged the RLS of IMR-90 by 19.7 PD at 100 *μ*M. Another product of PBN decomposition, benzaldehyde, was without effect or toxic at high concentrations. Other hydroxylamines, *N*-benzyl hydroxylamine and *N*-methyl hydroxylamine, delayed the senescence of IMR-90 cells by at least 17–20 PD. A simultaneous treatment of the cells with all three *N*-hydroxylamines (30 *μ*M each) yielded results similar to single treatments, delaying senescence by 14–17 PD. In contrast, the isomeric *O*-hydroxylamines at concentrations equivalent to the *N*-hydroxylamines either were without effect (*O*-*t*-butyl hydroxylamine) or induced a small decline in the final number of PDs (*O*-benzyl hydroxylamine and *O*-methyl hydroxylamine) [30].

Although nicotinamide and *N-t*-butyl hydroxylamine caused a similar level of lifespan extension in human fibroblasts, their action mechanisms may not be the same. The magnitude of the effect of nicotinamide was dependent on the duration of the treatment, and even after nicotinamide was removed, the population doubling continued for a further 20 or so PDs. In the case of *N-t-*butyl hydroxylamine, the magnitude of the effect was not dependent on treatment duration, nor was it cumulative. The lifespan gains were more prominent when they were applied to cells close to senescence. The effect of nicotinamide is also different from that of carnosine [66] and other antioxidant chemicals. Although carnosine had a pronounced effect in preventing senescent morphology, cells quickly entered senescence state upon its removal, neither did its effect appear to be cumulative. These differences suggest that the effect of nicotinamide is not a direct antioxidant effect [26].

*Methylene Blue* (MB) is a diaminophenothiazine that has been in clinical use for approximately 100 years in the treatment of a variety of ailments. Methylene Blue treats congenital and poison-induced methemoglobinemia, prevents the side effects of chemotherapy, and treats septic shock. There is weak evidence that chronic MB administration (2.5 mg/kg/day) extends the lifespan of mice. Methylene Blue was found to extend RLS of IRC-90 fibroblasts by >20 PD, 100 nM MB being more effective than 10 nM and 1 *μ*M stain. Interestingly, under 5% oxygen, 1 *μ*M MB was nearly as efficient as the 100 nM MB. Methylene Blue delayed cell senescence when administered at any PDL; however, the efficiency decreased as the starting PDL increased. The effect of MB was enhanced by simultaneous addition of 10 *μ*M or 100 *μ*M *N-t*-butyl hydroxylamine (increase of RLS by 30 PD; 100 nM alone: 21.5 PD). Thionine delayed cell senescence, though at higher concentrations than MB (1 *μ*M). Compounds with chemical structures similar to MB: 1,9-dimethyl-MB, Toluidine Blue O, and Celestin Blue were without effect. Methylene Blue increased the rate of cellular respiration, but this effect was indirect, mediated by changes in cellular metabolism. Dose dependently, MB induced or inhibited biosynthesis of Complex IV, heme synthesis and iron uptake, and increased ROS production measured with DCFDA [71]. Methylene Blue enhanced also fibroblast growth and induced Nrf2 and its downstream the antioxidant response element- (ARE-) response genes [72].

Fibroblasts used in studies of the effects of antioxidants on the RLS are listed in Table 1, and a summary of the results obtained is shown in Table 2.

## 4. Is the Action of Antioxidants due to Their Antioxidant Activity?

Compounds known as antioxidants have usually other physiological effects, indirectly related or not related at all to their antioxidant properties [77, 78]. Therefore, it should be taken into account that the effects of antioxidants on the RLS of fibroblasts may be due not (only) to their direct antioxidant actions but to their other biological activities.

Activation of sirtuins extends the lifespan and promotes longevity and healthy aging. Sirtuins are a family of NAD^+^-dependent enzymes that deacetylate substrates ranging from histones to transcriptional regulators, and they have been implicated in the molecular mechanisms of aging. It has also been reported that inhibitors of histone deacetylases accelerate cellular senescence [8]. Histone deacetylases are involved in DNA methylation and chromatin remodeling and thus in the modulation of gene expression [79]. SIRT-1, the mammalian ortholog of the yeast Sir2 protein, regulates, by means of deacetylation, the activity of proteins that play a crucial role in cellular senescence, such as p53 and the Forkhead Box O (FOXO) transcription factors [80, 81]. It was reported that the level of SIRT-1 decreases with serial cell passages as cells approach senescence [82]. Resveratrol was shown to be a sirtuin activator acting as an antiaging molecule in yeast cells [83]. This polyphenol has been shown to increase lifespan also in nematodes and fruit flies functioning as calorie restriction mimetic [84].

The proteasome is responsible for the removal of both normal and damaged proteins. Due to its latter function, the proteasome is also considered a main secondary antioxidant mechanism that becomes activated upon oxidative challenge. It is responsible for the cellular clearance from damaged and oxidized proteins that may cause deleterious side effects [85–87] upon lack of proteolysis and aggregation. It has been found that fibroblast cultures undergoing replicative senescence have reduced levels of proteasome activity accompanied by lower proteasome content due to the downregulation of its *β*-catalytic subunit [88]. The expression of proteasome subunits and the relative proteasome activities can be modulated by exogenous stimuli. The genes responsible for the formation of the 26S proteasome complex are coordinately regulated by Nrf2 in response to antioxidants [89]. More recently, Tan et al. evidenced that platelet-derived growth factor (PDGF) signaling and cytoskeletal structure can be dysregulated in senescent HDFs. The proproliferative effect of gamma-tocotrienol on senescent HDFs may be mediated through the stimulation of cellular response to stress and carbohydrate metabolism [74].

Aging is associated with progressive and site-specific changes in DNA methylation (DNAm). Fibroblasts mirror the established DNAm topology of the age-related elongation of very long-chain fatty acid (*ELOVL2*) gene in human blood and the rapid hypermethylation of its promoter *cg16867657*, which correlates with a linear decrease in *ELOVL2* mRNA levels across the lifespan. Using generalized additive modeling on twelve timepoints across the lifespan. Sturm et al. showed how single CpGs exhibit loci-specific, linear, and nonlinear trajectories that reach rates up to -47% (hypomethylation) to +23% (hypermethylation) per month. Together, these high-temporal resolution, global, gene-specific, and single CpG data highlight the conserved and accelerated nature of epigenetic aging in cultured fibroblasts, which may constitute a system to evaluate age-modifying interventions across the lifespan [90].

Fibroblast RLS was reported to be increased by cortisone [91]. While high doses of ionizing radiation are known to decrease RLS of fibroblasts, it was claimed that relatively low doses (3 × 1 Gy and 3 × 3 Gy) “can increase lifespan” of MRC-5 fibroblasts. However, this statement in the abstract of the paper is rather weakly supported by the reported data. Two cultures were irradiated 3 times with a dose of 1 Gy; the RLS of one was 65.9 PD, which is practically the same as that of control (66.1 PD); only for the second culture, RLS was prolonged (73.3 vs. 68.1 PD). Of course, the mean of two experiments pointed to an increase in RLS, but the reproducibility of this effect is far from satisfactory. A 3 × 3 Gy treatment lightly increased the RLS of cells in one culture (from 66.1 to 67.0 PD), while the RLS of the second culture was decreased (from 68.1 to 63.4 PD). While these data are not too convincing, the author reported that irradiated cultures had extended chronological time and some irradiated populations continued to grow long after controls had ceased cell division. The author hypothesized that multiple doses of *γ* rays may lead to the emergence of transformed lines from normal diploid human fibroblasts, which may have longer RLS or even be immortal, in analogy with induction of cancer by radiation. An alternative explanation was a cell response to stress induced by ionizing radiation [75]. In this context, it should be reminded that many antioxidants are known to autoxidize in cell culture media producing hydrogen peroxide, thus exerting a prooxidant effect [92–94]. It has been suggested in different contexts that the protective action of antioxidants can be partly due to their hormetic effect [95, 96]. Perhaps, a similar effect of antioxidants on the RLS of fibroblasts *in vitro* can be also ascribed, to some extent, to hydrogen peroxide produced by them or/and their other oxidation products.

## 5. Concluding Remarks

Fibroblasts are the cell type used as the first and most frequently to study aging at the cellular level *in vitro*. The interest in the effects of antioxidants on human lifespan and skin aging stimulated interest in studies of the effects of antioxidants on the RLS of fibroblasts *in vitro*.

Experiments on the *in vitro* effects of antioxidants may have limited relevance to the *in vivo* situation where high concentrations of antioxidants are usually not attainable and antioxidants are not so prone to autooxidation due to the lower oxygen concentration and more reducing environment. However, experiments on fibroblasts are more relevant to the applications of antioxidants in cosmetics as the exposure of antioxidants on or in the skin to oxygen may be comparable to that under conditions of *in vitro* cell culture, so the results of *in vitro* experiments on fibroblasts aging may be of relevance for the design of skin protecting preparations.

Evident from the presented data is the poor reproducibility of results between laboratories, and sometimes even within a lab. In spite of high standardization of cell culture methods, there are some factors, which evade scrutinous control, one of them concerning the way of preparation of antioxidant solutions and the time of their existence in the culture medium. It may be one of the factors contributing to the variability of results.

While some studies failed to demonstrate any positive effects of the RLS of fibroblasts, most studies pointed to small effects (increase by several PD) and few reported a significant prolongation of RLS. Further studies are needed to confirm the results of the most effective antioxidants and provide a deeper insight into the mechanisms of their action.

## Figures and Tables

**Table 1:** Fibroblast lines used in RLS studies.

Fibroblasts	Origin	References
2BS	Human fetal lung diploid fibroblasts human, female	[58]
FB0603	Human diploid foreskin fibroblasts	[47]
H8F2p 25LM fibroblasts	Isolated from human ear	[73]
HDFs	Human dermal fibroblasts	[8, 56, 60–62, 74]
HE49	Human embryonic fibroblasts, normal	[37]
HEFs	Human embryonic fibroblasts, established from an abortus	[35]
HFF1 (ATCC® SCRC1041™)	Human foreskin, from a newborn male	[48, 66]
HFL1 (ATCC® CCL-153™)	Isolated from human fetal lungs, Caucasian	[57]
HGPS	Normal human skin fibroblast	[72]
Hs68 (ATCC® CRL-1635™)	Human skin (foreskin), from a newborn male, Caucasian	[46]
IMR-90 (ATCC® CCL-186™)	Human lung, female, 16 weeks of gestation, Caucasian	[12, 22, 30, 71]
MRC-5 (ATCC® CCL-171™)	Human lung, 14 weeks of gestation fetus, male, Caucasian	[44, 66, 75]
Normal diploid fibroblasts	Isolated from foreskin	[25, 26, 59]
Normal human skin fibroblasts		[64]
NYaKe	Normal human adult fibroblasts derived from a 23-year-old healthy male	[27]
Primary cultures of human dermal fibroblasts	Female	[65]
WI-38 (ATCC® CCL-75™)	Human fetal lung diploid fibroblasts, 3 months of gestation fetus, female, Caucasian	[33, 34, 54, 55, 58, 76]
WS3RGB	Fibroblasts obtained from a 42-year-old female Werner syndrome patient	[37]

**Table 2:** Effect of antioxidants on the fibroblast replicative lifespan *in vitro*.

Antioxidant	Cell line; concentration; effect on RLS	Other effects	Reference
1,9-Dimethyl-Methylene Blue, Toluidine Blue O, and Celestin Blue	IMR-90 fibroblasts; concentration not specified: no effect		[71]
18*α*-Glycyrrhetinic acid	HFL1 fibroblasts; 2 *μ*g/ml; increase in RLS (58.5 to 64.3 PD)	Decrease in ROS level, Nrf2-mediated proteasome activation	[57]
2,2,6,6-Tetramethylpiperidine-1-oxyl (TEMPO)	IMR-90 fibroblasts; 25 *μ*M: no effect; 100 *μ*M: decrease of RLS (8–9PD)		[30]
3,3,5,5-Tetramethyl-l-pyrroline N-oxide	IMR-90 fibroblasts; 500 *μ*M; slight increase in RLS (+1 PD)		[22]
3-Carbamoyl-2,2,5,5-tetramethylpyrrolidin-1-yloxy (3-CP)	IMR-90 fibroblasts; 25 *μ*M; no effect; 100 *μ*M: decrease of RLS (8–9PD)		[30]
4-Hydroxy-2,2,6,6-tetramethylpiperidine-1-oxyl (4-OH-TEMPO)	IMR-90 fibroblasts; 25 *μ*M: no effect; 100 *μ*M: decrease of RLS (8–9PD)		[30]
Ascorbic acid 2-phosphate	200 *μ*M: HE49 cells, increase in RLS by 8.5 PD (20%) and 7.1 PD (2% O_2_); NYaKe cells, increase in RLS by 3.5 PD; WS3RGB cells, increase in RLS by 5.5 PD	Reduction of the rate of telomere shortening; decreased ROS level under 20% oxygen but not under 2% oxygen	[37]
Benzaldehyde	IMR-90 fibroblasts; 100 *μ*M: no or toxic effect		[30]
Berberine	2BS and WI38 fibroblasts; 0.3125 *μ*g/ml: no effect on RLS reported	Enhanced proliferation of cells during seven days of incubation. 3.5% SA-*β*-gal-positive cells seen in PD30 2BS cells, vs. 60% in late PDL cells; in berberine-treated 2BS cells (PD45), the SA-*β*-gal-positive cell rate reverted to 16%, as compared to the control group (56.5%). Decreased level of p16; cyclin protein and cyclin-dependent kinases, such as cyclin D1 and CDK4 increased. Upregulated level of phosphorylated retinoblastoma protein (pRB)	[58]
Butylated hydroxytoluene	IMR-90 fibroblasts; 100 *μ*M: no increase of RLS		[22]
Carnosine	HFF1 cells; 20 mM: RLS increased by 7.4 and 9.4 PD; 30 mM: RLS increased by 3.0, 4.3 and 6.3 PD; 50 mM: RLS decreased. MRC-5 cells; 20 mM: RLS extension by 10 and 14 PD; 30 mM: RLS extension by 4 and 8 PD; 20 mM and 30 mM, introduced at 55 PD: RLS increase by 13.0 and 12.1 PD, respectively. HFF-1 cells; 20 mM D-carnosine: no effect	50 mM carnosine: more flat, spread-out appearance; maintaining nonsenescent morphology	[66]
*Chlorella vulgaris* extract	Human diploid fibroblasts (HDFs); no effect of RLS reported	Young HDFs: reduced expression of *SOD1*, *CAT*, and *CCS*. Senescent cells: increased expression of the *SOD2* and *MAPK14* genes, downregulation of *TP53* gene expression. Young and senescent HDFs: decreased expression of *CDKN2A* gene, increased expression of *MAPK14* gene	[62]
Curcumin	H8F2p 25LM fibroblasts; 1: decrease of RLS		Sadowska-Bartosz et al., unpublished
Cyanidin	WI-38 cells; concentration not specified: increase of RLS (from 64 to 68 when added at 26 PD, from 62 to 65 when added at 42 PD, and from 62 to 63 when added at 58 PD)		[54]
Epigallocatechin gallate	Human diploid fibroblasts (HDF); 12.5 *μ*M: effect on RLS not reported	Decrease of ROS level, increased mitochondrial potential, more intact mitochondrial DNA, elevated activities of antioxidative enzymes, more juvenile cell status	[53]
Ginsenoside Rg3(*S*)	HDFs maintained until different passages: PD 8-12 (young cells) and PD 34-36 (senescent cells). Senescent HDFs starved with serum-free DMEM overnight and then incubated in DMEM containing 10 or 30 *μ*M Rg3(*S*) or (*R*) for 48 h: no effect on RLS reported	Decrease in percentages of SA-*β*-gal staining cells in replicatively old HDFs. Reversal of the replicative senescence of HDFs: restoration of the ATP level and NAD^+^/NADH ratio in senescent HDFs. Recovering the cellular levels of ROS and the NAD^+^/NADH ratio in young HDFs treated with rotenone. Downregulation of phosphatidylinositol 3-kinase/Akt through the inhibition of mTOR by cell cycle regulators like p53/p21 in senescent HDFs. Activation of sirtuin 3/PGC1*α* to stimulate mitochondrial biogenesis. Rg3(*R*) did not alter the corresponding signaling pathways	[61]
Kinetin	Primary cultures of human dermal fibroblasts; 40-200 *μ*M: no effect on RLS	Cells retained some characteristic features of young cells	[65]
L-Sulforaphane	IMR-90 fibroblasts; 1 nM–10 *μ*M: no effect		[71]
Malvidin	WI-38 cells; concentration not specified: increase of RLS (from 64 to 67 when added at 26 PD, from 62 to 66 when added at 42 PD, and from 62 to 63 when added at 58 PD)		[55]
Methylene Blue	IMR-90 fibroblasts; 100 nM: extension of RLS by >20 PD	Increase in ROS generation; concentration-dependent changes in the synthesis of complex IV and heme and iron uptake	[71]
*N,N*′-Diphenyl-1,4-phenylenediamine	IMR-90 fibroblasts; 40 *μ*M: no increase of RLS		[21]
*N*-Acetylcysteine	IMR-90 fibroblasts; 2 mM: no increase of RLS		[22]
*N*-Benzyl hydroxylamine	IMR-90 fibroblasts; 100 *μ*M: RLS prolongation by at least 17–20 PD	Decrease in Rh123 accumulation, prevention of the age-dependent decline in the activity of aconitase	[30]
Nicotinamide	FB0603 fibroblasts; 0.5 and 5 mM: small increase in RLS (+2 PD)		[47]
Nicotinamide	IMR-90 fibroblasts; 3 mM: no significant effect at	Morphological rejuvenation of aged cells	[12]
Nicotinamide	NHFs; 5 mM from PD 30: significant extension of RLS	Decreased lipofuscin accumulation, decreased mitochondrial superoxide, decreased acidic *β*-galactosidase	[69]
Nitroso-*tert*-butane (tNB)	IMR-90 fibroblasts; 10 *μ*M: no effect		[30]
*N*-Methyl hydroxylamine	IMR-90 fibroblasts; 100 *μ*M: RLS prolongation by at least 17–20 PD	Decrease in Rh123 accumulation, prevention of the age-dependent decline in the activity of aconitase	[30]
N-*t*-Butyl hydroxylamine	IMR-90 fibroblasts; 10 *μ*M: RLS prolongation by 5.8 PD; 100 *μ*M: RLS prolongation by 19.7	Decrease in Rh123 accumulation, prevention of the age-dependent decline in the activity of aconitase	[30]
*O*-Benzyl hydroxylamine	IMR-90 fibroblasts; 100 *μ*M: small decrease in RLS		[30]
*O*-Methyl hydroxylamine	IMR-90 fibroblasts; small decrease in RLS at 100 *μ*M		[30]
*O*-*t*-Butyl hydroxylamine	IMR-90 fibroblasts; 100 *μ*M: no effect		[30]
Piper betle (PB)	Primary HDFs derived from foreskins of three different male subjects aged between 9 and 12 years after circumcision; 0.4 mg/ml: no effect on RLS reported	Improvement of cell proliferation of young (143%), presenescent (127.3%) and senescent (157.3%) HDFs. Increased expressions of *PRDX6*, *TP53*, *CDKN2A*, *PAK2*, and *MAPK14* in senescent HDFs. Modulation of the transcriptional profile changes in senescent HDFs. Increased expressions of *SOD1*, decreased expression of *GPX1*, *PRDX6*, *TP53*, *CDKN2A*, *PAK2*, and *MAPK14* in PB-treated senescent HDFs	[59]
Quercetin caprylate, conc.	HFL1 fibroblasts; concentration not specified (≤5 *μ*g/ml): a statistically significant increase in RLS, less than 5%	Maintenance of young morphology, delay in appearance of senescent phenotype, increase in growth rate; proteasome activation; morphological rejuvenation	[48]
Resveratrol	MRC-5 cells; 5 *μ*M: small increase in RLS (+2 PD)	Reduced DNA breaks, attenuation of age-related increase in nuclear size, reduction in the level of acetylated forms of H3 and H4 histones and p53	[44]
Resveratrol	FB0603 cells; 0.2 and 1 *μ*M: no effect on RLS		[47]
Resveratrol	H8F2p 25LM fibroblasts; 1 *μ*M: decrease of RLS		Sadowska-Bartosz et al., unpublished
Resveratrol, oxyresveratrol, acetyl-resveratrol	Hs68 cells; 1-20 *μ*M resveratrol, oxy-resveratrol, acetyl-resveratrol: decrease in RLS		[46]
Salicylic acid	IMR-90 fibroblasts; 200 *μ*M: no increase of RLS		[22]
Thionine	IMR-90 fibroblasts; 1 *μ*M: extension of RLS		[71]
Urolithin A	Human dermal fibroblasts; no effect of RLS reported	Significantly increased type I collagen expression and reduced matrix metalloproteinase 1 (*MMP-1*) expression; reduced intracellular ROS, partially due to activation of the Nrf2-mediated antioxidative response. 50 *μ*M: changes in cell morphology and inhibition in cell proliferation, due to cell cycle arrest in G2/M phase. SA-*β*-gal and *γ*H2AX unaltered	[56]
Vitamin C (ascorbic acid)	HEFs; extension of RLS by 2 PD (2 *μ*M), 6 PD (20 *μ*M), and 11 PD (200)	Decreased SA-*β*-gal staining, decreased ROS level, increase in aconitase activity, decrease in of p53, phospho-p53 at Ser15and p21 and apurinic/apyrimidinic site level, relieve of senescence-related G1 arrest	[35]
Vitamin E	WI-38 cells; 10 *μ*g/ml and 100 *μ*g/ml: increase in RLS (from 65 to 109 and 115, resp.)	Size and shape typical of replicatively young cells, decreased autofluorescence	[33]
Vitamin E	WI-38 cells; 10 *μ*g/ml, 50 *μ*g/ml and 100 *μ*g/ml: no effect on RLS		[34]
*α*-Phenyl-*t*-butyl nitrone (PBN)	IMR-90 fibroblasts; 200 *μ*M: increase in RLS from 56.8 ± 5.1 PD to 60.5 ± 5.4 PD		[22]
*α*-Phenyl-*t*-butyl nitrone (PBN)	IMR-90 fibroblasts; 200 *μ*M: RLS prolongation by 2.4 PD; 800 *μ*M: RLS prolongation by 14.8 PD		[30]
*α*-Tocopherol acetate	IMR-90 fibroblasts; 200 *μ*M: no increase of RLS		[22]

## Data Availability

Data are available on request. Please contact I. Sadowska-Bartosz (e-mail: isadowska@poczta.fm).

## Abbreviations

BMEM: Basal Modified Eagle's Medium
DCFDA: 2′,7′-Dichlorofluorescin diacetate
DMEM: Dulbecco's Modified Eagle Medium
ECM: Extracellular matrix
EGCG: Epigallocatechin gallate
HDAC: Histone deacetylase
HSFs: Human skin fibroblasts
MEM: Eagle Minimal Essential Medium
N-HDAC: Nicotinamide adenine dinucleotide- (NAD^+^-) dependent histone deacetylase
PD: Population doubling
Rh123: Rhodamine 123
RLS: Replicative lifespan
ROS: Reactive oxygen species
SA-*β*-gal: Senescence-associated *β*-galactosidase
SIRT: Sirtuin
SOD: Superoxide dismutase
TEMPO: 2,2,6,6-Tetramethylpiperidine-1-oxyl
TEMPOL: 4-Hydroxy-2,2,6,6-tetramethylpiperidine-1-oxyl.
